# Comprehensive Analysis of Placental DNA Methylation Changes and Fetal Birth Weight in Pigs

**DOI:** 10.3390/ijms25147702

**Published:** 2024-07-13

**Authors:** Baohua Tan, Liyao Xiao, Yongzhong Wang, Chen Zhou, Huijun Huang, Zicong Li, Linjun Hong, Gengyuan Cai, Zhenfang Wu, Ting Gu

**Affiliations:** 1 State Key Laboratory of Swine and Poultry Breeding Industry, National Engineering Research Center for Breeding Swine Industry, College of Animal Science, South China Agricultural University, Guangzhou 510642, China; tanbaohua@stu.scau.edu.cn (B.T.); xlyao@stu.scau.edu.cn (L.X.); yz_wang@stu.scau.edu.cn (Y.W.); 9906602@haust.edu.cn (C.Z.); 1227704881@stu.scau.edu.cn (H.H.); lizicong@scau.edu.cn (Z.L.); linjun.hong@scau.edu.cn (L.H.); cgy0415@scau.edu.cn (G.C.); wzf@scau.edu.cn (Z.W.); 2 Guangdong Provincial Key Laboratory of Agro-Animal Genomics and Molecular Breeding, College of Animal Science, South China Agricultural University, Guangzhou 510642, China

**Keywords:** pigs, DNA methylation, placental development, birth weight

## Abstract

Birth weight is a complex multifactorial trait relevant to health states and disease risks in later life. The placenta is essential for proper fetal growth and facilitates gas, nutrient, and waste exchange between the mother and developing fetus. How changes in placental DNA methylation affect fetal birth weight remains to be fully elucidated. In this study, we used whole-genome bisulfite sequencing and RNA sequencing to reveal a global map of DNA methylation and gene expression changes between the placentas of highest birth weight and lowest birth weight piglets in the same litters. The transcriptome analysis identified 1682 differential expressed genes and revealed key transcriptional properties in distinct placentas. We also identified key transcription factors that may drive the differences in DNA methylome patterns between placentas. The decrease in DNA methylation level in the promoter was associated with the transcriptional activation of genes associated with angiogenesis, extracellular matrix remodeling, and transmembrane transport. Our results revealed the regulatory role of DNA methylation in gene transcription activity leading to the differences in placental morphological structures and birth weights of piglets. These results could provide novel clues to clarify the underlying regulatory mechanisms of placental development and fetal growth.

## 1. Introduction

The birth weight of newborns reflects not only the development of the fetus in the maternal environment but also the nutritional and metabolic status of the mother [1]. For survival, the fetus may slow down its metabolism and growth to adapt to the intrauterine environment, which would cause changes in physiology, structure, and metabolism, resulting in long-term negative consequences [2]. Previous studies have shown that low birth weight is consistently associated with higher blood pressure, insulin resistance, type 2 diabetes, and cardiovascular disease [3,4,5,6,7]. It is also commonly recognized that a low birth weight is associated with a low survival rate, poor growth performance, and poor meat quality in pigs [8,9]. Thus, clarifying the regulatory mechanisms of birth weight is important to reduce the risk of disease in later life and for improved animal production. 

The placenta, having endocrine and material transport functions, is an essential transient organ at the maternal–fetal interface, providing nutrition for fetal growth. Placental development is greatly associated with the parturition process, a reproductive trait of sows [10,11]. Appropriate nutrient provision to the fetus, via the placental blood, is essential for optimal fetal growth [12]. Increased placental efficiency contributes to a large litter size in sows [13]. Having a high-weight placenta could cause adverse perinatal outcomes, such as fetal respiratory distress syndrome and death, while having a low-weight placenta is related to maternal complications and low birth weight [14]. There are various factors that determine the ability of the placenta to transport nutrition to the fetus [15,16]. DNA methylation mainly occurs via DNA methyltransferases (DNMTs) transferring a methyl group to the fifth carbon position of the cytosine in the CpG dinucleotide [17] and inhibits gene expression by preventing transcription factors or recruiting methyl-binding proteins [18]. It has been demonstrated that DNA methylation regulates the trophoblast invasion and placental development that is important for fetal growth [19,20], but how DNA methylation functions in shaping placental morphological structures and controlling the birth weight of piglets remains largely unknown. 

Our previous study revealed DNA methylome profiling in placentas from pig fetuses at 21, 28, and 35 days post-coitus [21]. In this study, we first integrated RNA sequencing (RNA-seq) and whole-genome bisulfite sequencing (WGBS) to provide a comprehensive analysis of gene expression and DNA methylation patterns in placentas from piglets with different birth weights in the same litters. We highlighted the critical role of DNA methylation in shaping transcription programs in distinct placentas. This study could provide novel insights into the involvement of DNA methylation in the regulation of placental development and the birth weight of piglets.

## 2. Results

### 2.1. Morphology and Gene Expression Changes between the HBW and LBW Placentas

**Figure 1:**
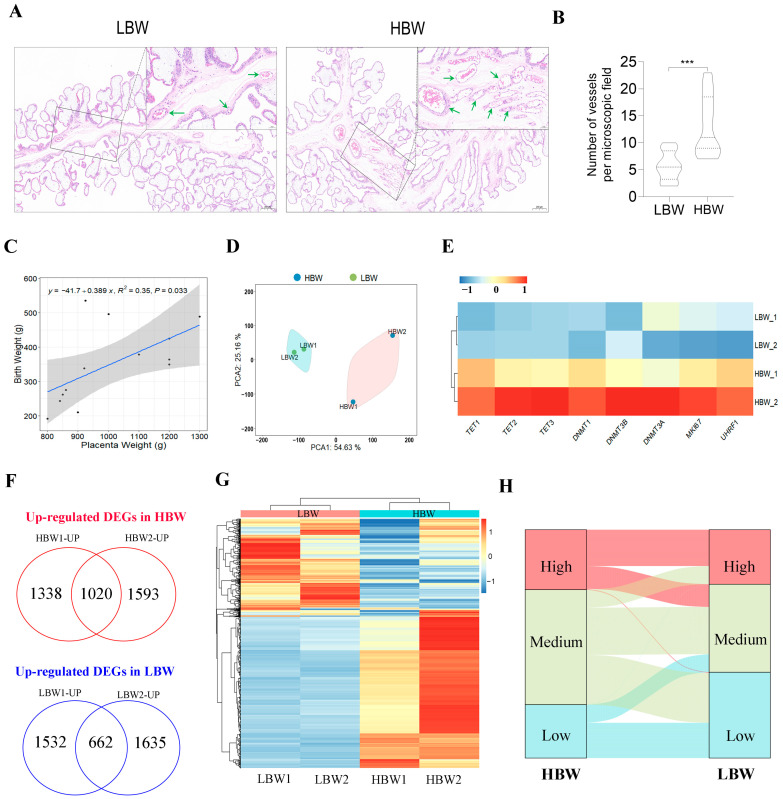
Morphology and gene expression changes between the HBW and LBW placenta. (**A**) H–E staining, morphological changes in HBW and LBW placentas with the placenta seen in 5× magnification and in further detail at 20× magnification (upper-right panels). The green arrows indicate the placental vessels. Scale bar, 200 μm and 50 μm (upper-right panels). (**B**) Histograms indicating placental vascular density in each group. *** *p* < 0.001. (**C**) Scatter plot showing the positive correlation between birth weight and placenta weight (*n* = 13). The blue line represented the fitted linear regression model to each sample. The gray highlight indicated the 95% confidence interval. (**D**) The results of PCA on gene expression data in each sample. (**E**) The heatmap of RNA-seq-based expression levels of DNMTs and dMTases. Different colored circles indicated the 95% confidence interval. (**F**) The overlapping of up-regulated DEGs or down-regulated DEGs between the LBW group and HBW groups. (**G**) The hierarchical clustering heatmap of DEGs. (**H**) The alluvial diagram of DEGs. High (TPM ≥ upper quartile); medium (lower quartile < TPM < upper quartile); low (TPM ≤ lower quartile).

In this study, we collected placenta samples from piglets with the highest birth weight (HBW) and lowest birth weight (LBW) in the same litter (Supplementary Table S1). The HBW and LBW placentas were both from female fetuses to avoid the effects of gender differences. The morphological characteristics of porcine placental tissues were microscopically compared via hematoxylin–eosin (H–E) staining (Figure 1A). The HBW placenta showed increased nucleated red blood cells within the vessels and intervillous space than the LBW placenta (Figure 1B). We found a positive correlation between the birth weight and placenta weight, suggesting the importance of the placenta size in fetal growth (Figure 1C). We then performed RNA-seq to reveal the transcriptome dynamics in HBW and LBW placentas. Approximately 171 million reads with a unique mapped rate of above 89% were obtained (Supplementary Table S2). The principal component analysis (PCA) results showed a closed clustering of biological replicates, indicating the high reproductivity of the biological samples (Figure 1D). We found higher expression rates of DNA methylases (DNMTs) and DNA demethylases (dMTases) on the HBW placenta than on the LBW placenta, which was consistent with the changes in the expression of Ki67, the proliferation marker protein in trophoblast cells (Figure 1E). These results suggested that the process of DNA methylation might have been more activated in the HBW placenta than the LBW placenta. In total, we identified 1020 up-regulated differentially expressed genes (DEGs) and 662 down-regulated DEGs when comparing the HBW placenta to the LBW placenta (Figure 1F) (Supplementary Table S3). The hierarchical clustering with heatmaps showed similar expression patterns between the placentas from the same group (Figure 1G). The alluvial diagram was generated to show the degrees of differences in the expression of DEGs (Figure 1H). There were seven DEGs with dramatic changes in expression, such as the leading imprinted gene *PEG3*, which was significantly up-regulated in the HBW placenta. This result was consistent with a previous study that showed that the disruption of *PEG3* contributed to small placentas and embryos with asymmetric intrauterine growth restrictions [22]. Collectively, we revealed the differences in morphological characteristics and gene expression patterns in the HBW and LBW placentas. 

### 2.2. Functional Enrichment Analysis of Differentially Expressed Genes

**Figure 2:**
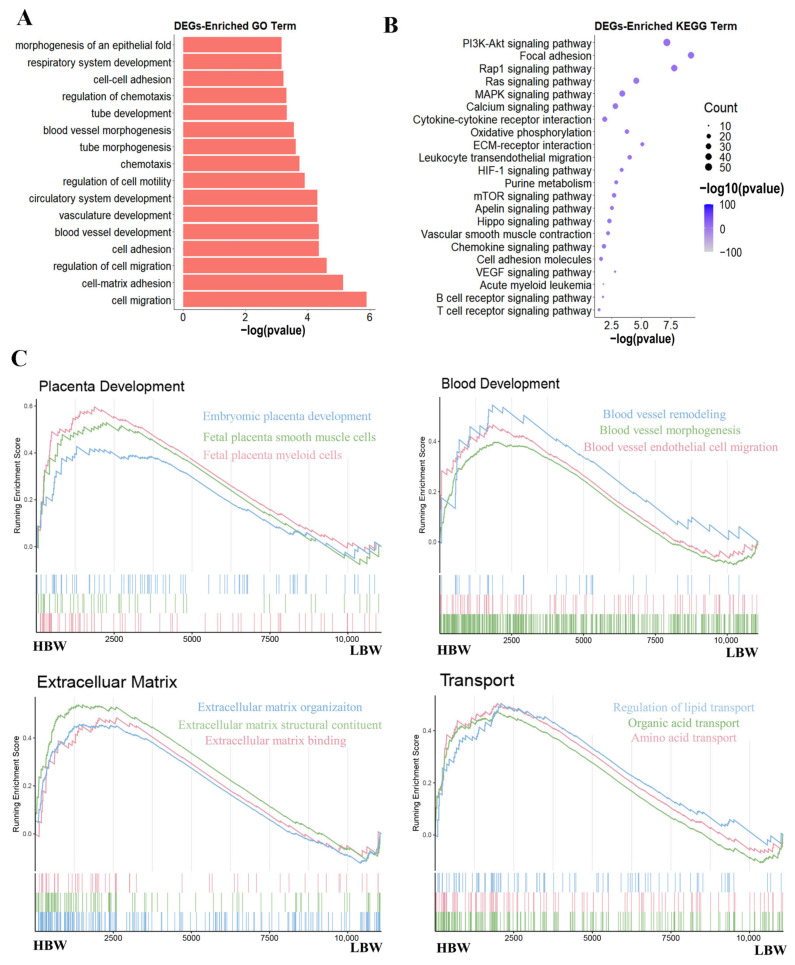
Functional enrichment analysis of differentially expressed genes. (**A**) The GO enrichment analysis of the DEGs. (**B**) The KEGG pathway analysis of DEGs. (**C**) GSEA plot of genes involved in various processes.

To explore the function of DEGs, we subjected the DEGs to gene ontology (GO) and Kyoto Encyclopedia of Genes and Genome (KEGG) pathways analysis (Supplementary Table S4). GO analysis indicated the significant enrichment of blood development-related and chemotaxis-related processes in DEGs (Figure 2A). KEGG enrichment analysis showed the enrichment of metabolism-related pathways, such as MAPK signaling and oxidative phosphorylation; and oxygen homeostasis-related pathways, such as PI3K/Akt and HIF-1 signaling pathways (Figure 2B). Gene set enrichment analysis (GSEA) was performed to investigate differences in biological processes associated with placental development between the HBW and LBW placentas. Our results showed that the processes of placenta development, blood development, extracellular matrix (ECM), and transport activity were more activated in the HBW placenta (Figure 2C). In summary, these results revealed the dynamic differences in transcriptome properties in the HBW and LBW placentas.

### 2.3. Characteristics of DNA Methylome in the HBW and LBW Placentas

**Figure 3:**
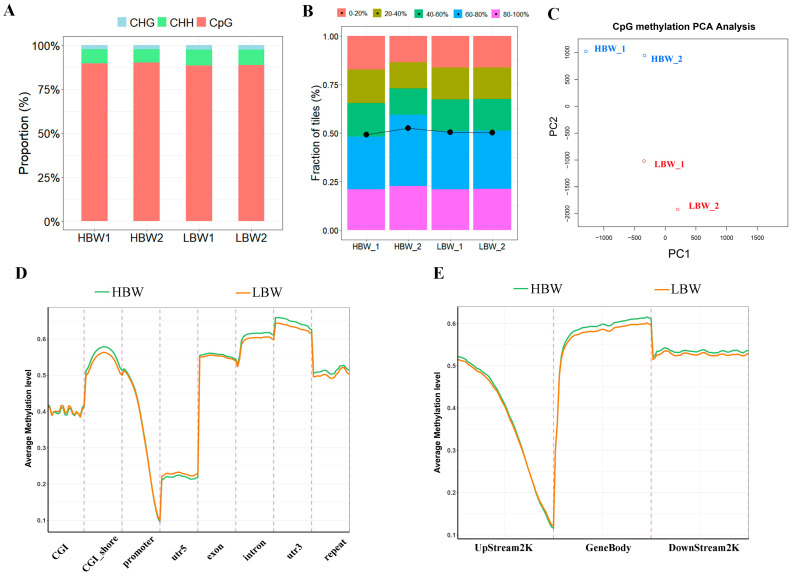
Characteristics of DNA methylome in the HBW and LBW placentas. (**A**) The proportion of methylated cytosine sites in CHG, CHH and CpG (where H = A, C, or T). (**B**) Fraction of total CpGs with different methylation levels in distinct placentas. The (methylated CpG)/(all CpG) ratio, from left to right, 0.492708, 0.525829, 0.505327, 0.502685. (**C**) The results of PCA on CpG methylation in each sample. (**D**,**E**) The average methylation level of CpGs in different genomic features between the HBW and LBW placentas.

Next, we performed WGBS to characterize the DNA methylation profiles in the HBW and LBW placentas. We obtained approximately 1.22 billion paired-end reads. The average mapping rate of clean reads mapping to the Sscrofa 11.1 reference genome was 81.35% (range 80.79% to 81.81%) (Supplementary Table S2). As the methylated cytosine sites across different samples predominantly occurred in a CpG sequence context (approximately 80%) (Figure 3A), we focused on CpG methylation in the further analysis. All the samples had similar CpG methylation levels (approximately 60%), and most CpGs were heavily methylated (Figure 3B). The PCA results revealed a clear separation of the HBW and LBW groups, and two samples from each group were clustered (Figure 3C). We compared the methylation levels in different genomic features between the HBW and LBW placentas. The methylation level of CpGs was notably low in the promoter and 5′ untranslated regions (UTR), while heavily methylated CpGs were found in the CGI shore 3′ UTR and exon (Figure 3D,E). These results showed the characteristics of DNA methylation in HBW and LBW placentas.

### 2.4. Dynamic Changes in DNA Methylation Landscape in the HBW and LBW Placentas

**Figure 4:**
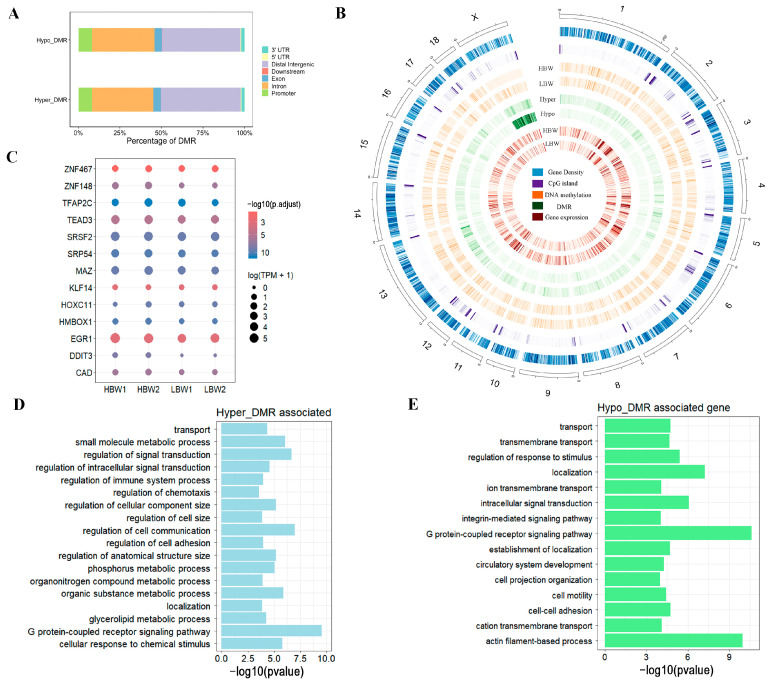
Dynamic changes in DNA methylation landscape in the HBW and LBW placentas. (**A**) The proportion of DMRs on different genomic features. (**B**) Circos analysis of the methylation distribution on all chromosomes. From outermost ring to innermost ring: (1) Gene density. (2) CpG island. (3) Mean methylation level of the HBW placenta. (4) Mean methylation level of the LBW placenta. (5) Hyper-methylated DMRs. (6) Hypo-methylated DMRs. (7) Mean methylation level of HBW placenta. (8) Mean methylation level of LBW placenta. (**C**) TF enrichment analysis of DMRs in the promoter. (**D**,**E**) GO analysis of hyper- and hypo-aaDMRs across the promoter.

To explore the function and regulatory mechanism of DNA methylation in placenta development, we identified differentially methylated regions (DMRs) and transcription factors between the HBW and LBW placentas (Supplementary Table S5). We obtained 34,641 hypermethylated DMRs (referred to as hyper-DMRs) and 24,517 hypomethylated DMRs (referred to as hypo-DMRs) compared to the HBW placenta and the LBW placenta, respectively. As a result, we observed that DMR regions were mainly located in the distal region and intron and identified about 9.18% of all DMR regions occurring in the gene promoter (Figure 4A). A Circos plot was used to examine the distribution of DMRs in the genome. We found that the DMRs were present on all chromosomes (Figure 4B). To identify the key transcription factor that is associated with DNA methylation levels, we used Homer software (version 4.10) to perform motif analysis for DMRs in the promoter (promoter-DMRs). The results showed that promoter-DMRs were significantly enriched in the binding motifs of placenta-related transcription factors (TFs), including TFA2PAC and EGR1 (Figure 4C). GO analysis revealed that the genes with hypermethylated promoter-DMRs in the promoter were enriched in the regulation of anatomical structure size, transport, metabolism, and signal transduction (Figure 4D), while the genes with hypomethylated promoter-DMRs were associated with the processes of transmembrane transport, localization, and circulatory system development (Figure 4E). These results suggested the important role of DNA methylation in regulating the transcription activity of genes in distinct placentas and identified a set of placenta-related TFs. 

### 2.5. Association between DNA Methylation and Gene Expression in the HBW and LBW Placentas

**Figure 5:**
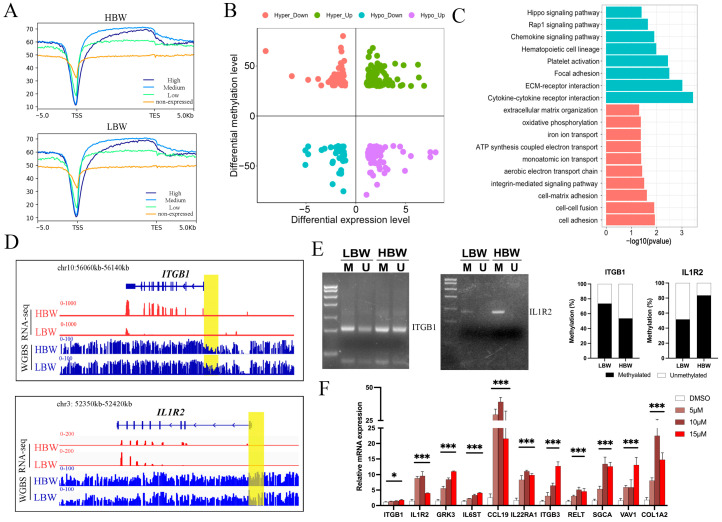
Integrative analysis of DNA methylome and transcriptome in the placenta. (**A**) The enrichment plot showing the DNA methylation levels around gene bodies of genes with different expression levels (high, medium, low, and non-expressed). High (TPM ≥ upper quartile; medium (lower quartile < TPM < upper quartile); low (TPM ≤ lower quartile); non-expressed (TPM < 1). (**B**) Four-quadrant plot showing the genes with significant changes in both methylation levels and expression level in the HBW and LBW placentas. Different types of genes were represented in different colors. (**C**) The GO and KEGG enrichment analysis of PNGs. Blue: KEGG pathway; Red: GO biological process. (**D**) IGV visualization of the tracks of WGBS and RNA-seq between *ITGB1* and *IL1R2* gene in HBW and LBW placentas. DMRs in the promoter is colored in yellow. (**E**) MSP analysis of the promoter of *ITGB1* and *IL1R2* in HBW and LBW placentas. M: methylated alleles. U: unmethylated alleles. (**F**) The mRNA expression changes in selected PNGs following 5-Aza-induced demethylation in PTr2 cells. * *p* < 0.05 *** *p* < 0.001.

As a well-known epigenetics factor, the dynamic changes in DNA methylation are closely related to gene repression [18]. To examine the relationship between DNA methylation and gene expression level, we divided all expressed genes into four classes based on the expression levels (high, medium, low, and non-expressed) (Figure 5A). The enrichment profile results showed that the gene expression and methylation levels of the gene promoter were negatively correlated (Figure 5B). Thus, we combined the analysis results of the DNA methylome and transcriptome to identify DEGs that were regulated by DNA methylation. As a result, we obtained 330 DEGs with at least a promoter-DMR, among which 104 DEGs were denoted as promoter-negative genes (PNGs), including 47 down-regulated DEGs with hypermethylated DMRs and 58 up-regulated DEGs with hypomethylated DMRs. GO and KEGG analysis revealed that these PNGs were enriched in placental development-related pathways, including chemokines signaling, extracellular matrix organization, integrin-mediated signaling, oxidative phosphorylation, and Rap1 signaling (Figure 5C; Supplementary Table S6). Differential expression levels and DNA methylation patterns of *ITGB1* and *IL1R2* in HBW and LBW placentas were shown by the Integrative Genomics Viewer (IGV) (Figure 5D). Methylation-specific PCR (MSP) experiments were performed to further validate the differential methylation levels of *ITGB1* and *IL1R2* in the HBW and LBW placentas (Figure 5E). To investigate the effect of DNA methylation on PNGs, porcine trophoblast cells (PTr2) were treated with 5-Aza-2′-deoxycytidine (5-Aza), a DNMTs inhibitor that causes alterations in DNA methylation [16]. The results showed that the mRNA expression level of selected PNGs was significantly upregulated following 5-Aza-induced demethylation (Figure 5F). Collectively, our study identified a set of placental development-related genes that are regulated by DNA methylation. DNA methylation could regulate placental development by mediating gene repression, which might ultimately lead to the differences in piglets’ birth weight. 

## 3. Discussion

The insufficiency of placental metabolic and endocrine function caused by disturbances of placental gene expression contributes to low fetal birth weight and poor growth, which severely affects normal fetal development and homeostasis and leads to increased fetal morbidity, slower growth rates, and greater mortality [23,24]. Previous studies have revealed that DNA methylation plays critical roles in the expression patterns of genes associated with placenta formation and function [19,20,21], but it has not been connected to fetal development. In this study, we compared the microstructural structure and DNA methylome pattern of the HBW and LBW placentas and analyzed the corresponding transcriptome characteristics. 

The normal development of placental vessels is essential to ensure the exchange of oxygen and nutrients needed for fetal growth [25]; thus, placental vascular dysplasia results in fetal developmental defects and even intrauterine embryonic mortality [26]. In this study, H–E staining results showed great differences in the placental vessel structure between the HBW and LBW placentas. Specifically, we found that the HBW placentas showed more nucleated red blood cells within the vessels and intervillous space than the LBW placenta, suggesting the greater ability to exchange oxygen and nutrients necessary for fetal growth in HBW piglets. Consistent with this finding, the functional enrichment analysis of DEGs was enriched in the pathways related to vasculature development, such as blood vessel morphogenesis and the VEGF signaling pathway. Moreover, GSEA showed that the process of blood development is more activated on the HBW placentas than on the LBW placentas. Particularly, the expression levels of vascular endothelial growth factor A (VEGFA) and receptor 2 (VEGFR-2) were higher in the HBW placentas than the LBW placentas, suggesting activated angiogenesis and vascular permeability in the HBW placenta [27]. These results highlighted the differences in blood development between the HBW and LBW placentas, which might be an important factor that causes the different birth weights of piglets. 

The DNA methylome in the HBW and LBW placentas was compared to further investigate the function of DNA methylation in shaping tissue-specific transcription patterns. Our results suggested that DMRs widely existed in HBW and LBW placentas and were closely associated with the altered expression of placental development-related genes. We found that several reported placenta development-related TFs were enriched in the DMRs and potentially modulate DNA methylation levels in placentas, such as TFAP2C, which was required for trophoblast stem cell maintenance and embryonic implantation [28]. It was consistent with a previous finding, which reported that TFAP2C can regulate DNA methylation levels in porcine early embryonic development [29]. Collectively, our results identified a set of candidate placental development-related TFs, which have important implications for elucidating differences in placental DNA methylation between the HBW and LBW fetuses. 

DNA methylation is important to repress gene expression by altering chromatin accessibility and affecting the occupation of transcription factors. In this study, we found that DNA methylation exhibited a significantly negative correlation with gene expression in the corresponding promoter at the global level. We finally identified 104 PNGs that were enriched in the process of placental development, such as cytokine–cytokine receptor interaction, RAP1 signaling pathway, and ECM organization. The cell-to-ECM interactions are essential for proper placental development [30]. The Collagen type I alpha 2 chain (COL1A2) and Collagen type VII alpha 1 chain (COL7A1) are the crucial components of the ECM, which were up-regulated with decreased DNA methylation in HBW compared to LBW placenta. Rap1 signaling can promote vasculogenesis and angiogenesis [31]. Our results showed that some genes involved in the RAP1 signaling pathway were repressed by DNA methylation, including *VAV1*, *ITGB3*, and *ITGB1*. Cytokines can activate the transport of intracellular signals and induce epigenetic modification in fetuses, which can affect metabolic properties and the health state of offspring [32]. A previous study indicated that cytokines released from placentas may activate particular inflammatory pathways and promote maternal insulin resistance, which is essential for a normal pregnancy [32]. Cytokine IL-32 is silenced by DNA methylation in H3K293 cells [33]. In this study, we extended this finding and demonstrated that the expression of genes encoding the cytokines *IL-1R2* and *IL-6ST* was repressed by DNA methylation during placental development. Collectively, we identified a set of candidate genes regulated by DNA methylation. Our results indicated that DNA methylation is crucial for the formation of transcriptome patterns in the HBW and LBW placentas, primarily regulating the processes of ECM organization, blood development, and cytokine production.

The birth weight is greatly influenced by the expression of imprinted genes, such as the *IGF2* and *PHLDA2* genes [34,35]. Thus, the gender differences in fetuses might also affect fetal birth weight. In this study, we collected six HBW or LBW placentas in total from different litters. Our results showed that there was no obvious gender preference in the HBW and LBW fetuses. To decrease the effect of gender differences and litter size, we only chose two litters with similar size where the number of female and male fetuses was close, and both LBW and HBW fetuses were female fetuses for sequencing. The competition among fetuses is also the main cause of differences in birth weight. Taken together, it will be necessary to control the same size and gender distribution of litters and increase the number of sequencing samples in further studies. 

The pigs with a low birth weight exhibited a decreased percentage of muscle and an increased percentage of body fat at slaughter [36], which will cause great economic loss for the swine producer. Increasing the feed intake of sows in late gestation has been evidenced to increase piglet birth weight [37], while some studies have observed that maternal over-nutrition may harm fetal growth [38]. An alternative approach to reducing low-birth-weight offspring is genetic selection. Previous studies have identified some easily classified fitness traits that are suitable for direct selection, such as offspring vitality or vigor [39]. In this study, we found a positive correlation between birth weight and placenta weight. Thus, it will be a possible approach to use the information of placenta weight to apply in genetics selection for reducing low-birth-weight piglets.

## 4. Conclusions

Our study analyzed the genome-wide DNA methylation profiles of placentas corresponding with different birth weight piglets. The regulation of DNA methylation in gene transcription activity was associated with the differences in placental morphological structures and birth weight of piglets. These results could shed new light on clarifying the potential regulatory mechanisms of DNA methylation in placental development and fetal growth.

## 5. Materials and Methods

### 5.1. Sample Collection

All third-parity Duroc sows were managed in the same environmental conditions and fed the same standard diet. The sows used for sequencing were half-siblings and mated naturally with the same boar after estrus. The piglets with the heaviest and lowest birth weight in the same litter were selected and defined as HBW and LBW piglets, respectively. Placenta samples of HBW or LBW piglets were excised from the maternal side of the placenta, 2 cm from the umbilical cord insertion site and free of maternal decidua corresponding to the chorioallantoic placenta [21]. The collected placental tissues were rapidly placed in liquid nitrogen.

### 5.2. Cell Culture and 5-Aza Treatment

PTr2 cells were isolated from early embryos of sows at day 12 of pregnancy and culture with 5% CO_2_ at 37 °C as reported in previous study [40]. DMEM/F12 basic medium (Gibco, Grand Island, NY, USA) supplemented with 10% fetal bovine serum (Gibco, Grand Island, NY, USA), 0.5% insulin (YEASEN, Shanghai, China), and 1% penicillin-streptomycin (Gibco, Grand Island, NY, USA) was used for PTr2 cells culture. For DNA methylation inhibition, PTr2 cells were treated with 5, 10, and 20 μmol/L (μM) 5-Aza (Sigma-Aldrich, Saint Louis, MO, USA) for 5 days. The medium was replaced every 24 h. 

### 5.3. Whole Genome Bisulfite Sequencing and Data Analysis

The genomic DNA was extracted from placenta samples and then was fragmented to 200–300 bp by using Covaris S220 (Covaris, Woburn, MA, USA). After purification, the DNA fragments were added with adenosine in the 3′ ends and connected with an adaptor. Adaptor-ligated DNA fragments were treated with bisulfite and contrasted to a sequencing library according to the manufacturer’s instructions. The library was finally sequenced on the Illumina Novaseq platform (150 bp paired-end) (Illumina, San Diego, CA, USA). The adapter in reads and low-quality reads of raw data were trimmed to generate clean data by using trim_galore (https://www.bioinformatics.babraham.ac.uk/projects/trim_galore/ accessed on 3 February 2024). The clean reads were mapped to pig reference genome by using Bismark (version 0.19.0) with default parameters [41]. The number of methylated cytosine divided by the sum of methylated cytosine and unmethylated cytosine at each CpG site was defined as DNA methylation level. DMR regions were identified by R package MethyKit [42]. The annotation of DMR regions was performed by the R package ChIPseeker [43]. IGV software (version 2.10) was used to visualize the DNA methylation and expression patterns in selected regions [44].

### 5.4. RNA-Seq and Data Analysis 

Total RNA was extracted from placenta samples by using TRIzol reagent (Gibco, Grand Island, CA, USA). The purified RNA was then subjected to library construction by using NEBNext R UltraTM RNA Library Prep Kit for Illumina R (New England BioLabs, Ipswich, MA, USA). The prepared library was sequenced on the Illumina NovaSeq 6000 platform (150 bp paired-end). Fastq (version 0.23.1) was used to remove adapter and low-quality reads of raw sequencing [45]. Hisat2 (version 2.2.1) was used to build genome index and mapped clean reads to the reference genome of pig (susScr11) [46]. The mapped reads count of genes were quantified by using featureCounts (version 2.0.1) [47]. The raw genes count was normalized to the transcripts per kilobase million (TPM) by using custom scripts. The R package DESeq2 was used to perform differential gene expression analysis with the criteria log2 fold-change > 1 and FDR < 0.05 [48]. GO analysis was performed in PANTHER [49]. KEGG pathway analyses were carried out in KOBAS-i [50]. GSEA were implemented and visualized using the R package GseaVis (https://github.com/junjunlab/GseaVis accessed on 3 February 2024). The circos plot of DMRs was generated by Circos software (version 0.67) [51]. 

### 5.5. Real-Time Quantitative PCR (qPCR)

Total RNA was extracted and then reverse transcribed into cDNA by using Evo M-MLV RT Kit (AG, Changsha, China). qPCR experiment was performed by using PowerUp^TM^ SYBR^TM^ Green Master Mix (Thermo Fisher Scientific, Waltham, MA, USA). The relative gene expression level was calculated using the Ct (2^−ΔΔCt^) method. All primers used for qPCR were shown in Supplementary Table S7.

### 5.6. Methylation-Specific PCR (MSP)

The genomic DNA of the HBW and LBW placentas was extracted and bisulfite-treated by using EZ DNA Methylation-Direct kit (Zymo Research, Irvine, CA, USA). The specific MSP primers designed by MethPrimer [52] were shown in Supplementary Table S7. The PCR products were subjected to 3% TAE agarose gel electrophoresis.

### 5.7. Statistical Analysis

The statistical analysis for the results of qPCR was performed by SPSS 20.0 software (IBM, Armonk, NY, USA) with an independent sample Student’s *t*-test. Experimental results were presented as the mean ± standard error of mean (SEM).

## Data Availability

The datasets presented in this study are deposited in the NCBI Sequence Read Archive (SRA), and the records can be accessed by accession numbers PRJNA1067378.

## Supplementary Materials

The following supporting information can be downloaded at: https://www.mdpi.com/article/10.3390/ijms25147702/s1.
