# Supplementary figures and images for: Comprehensive Analysis of Placental DNA Methylation Changes and Fetal Birth Weight in Pigs
Source: Int J Mol Sci. 2024 Jul 13;25(14):7702. doi: 10.3390/ijms25147702 (PMC11276634; doi:10.3390/ijms25147702)

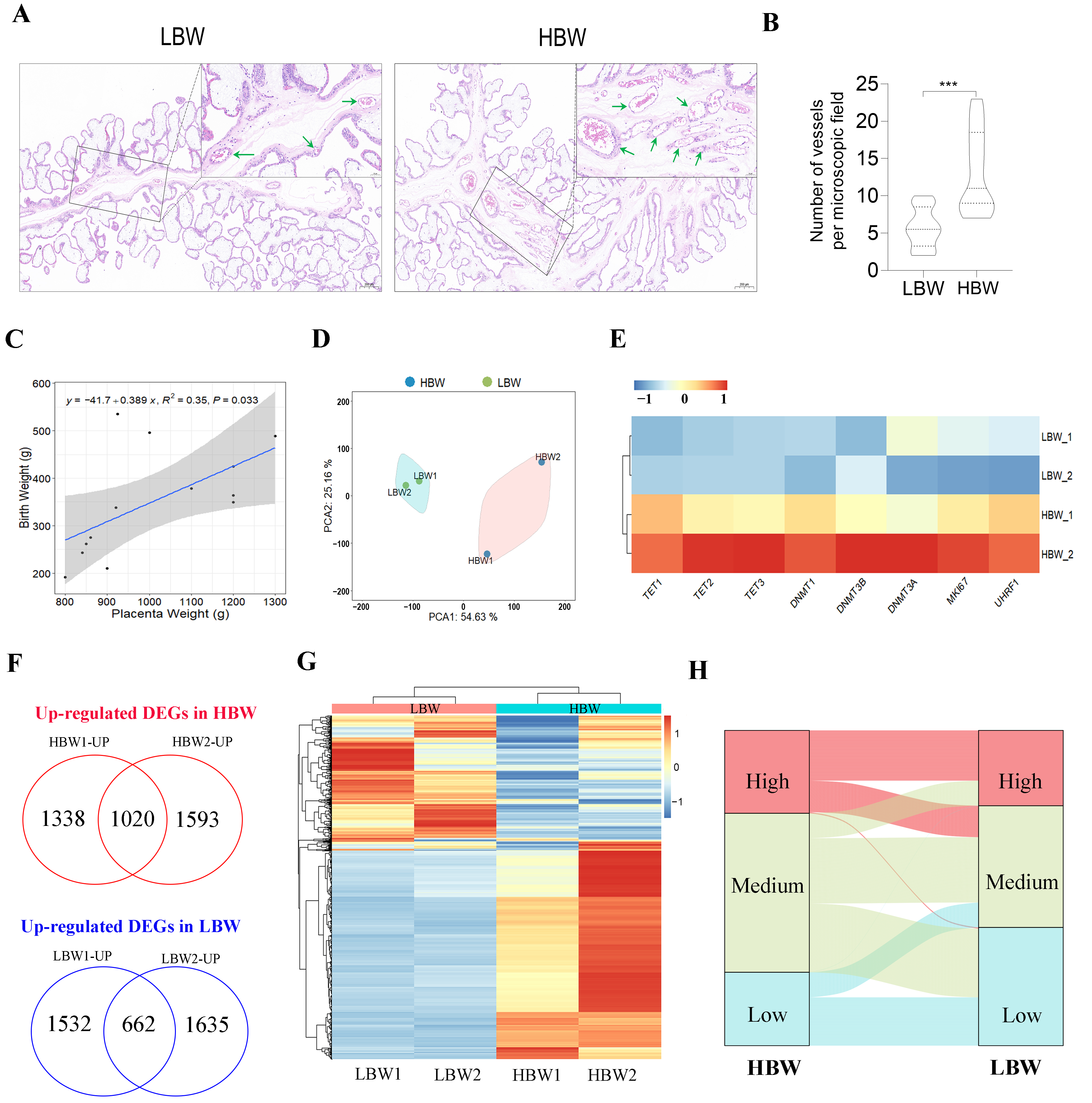

Supplement: Supplementary file 1 [file ijms-25-07702-s001.zip › Figure/Figure S1.tif]

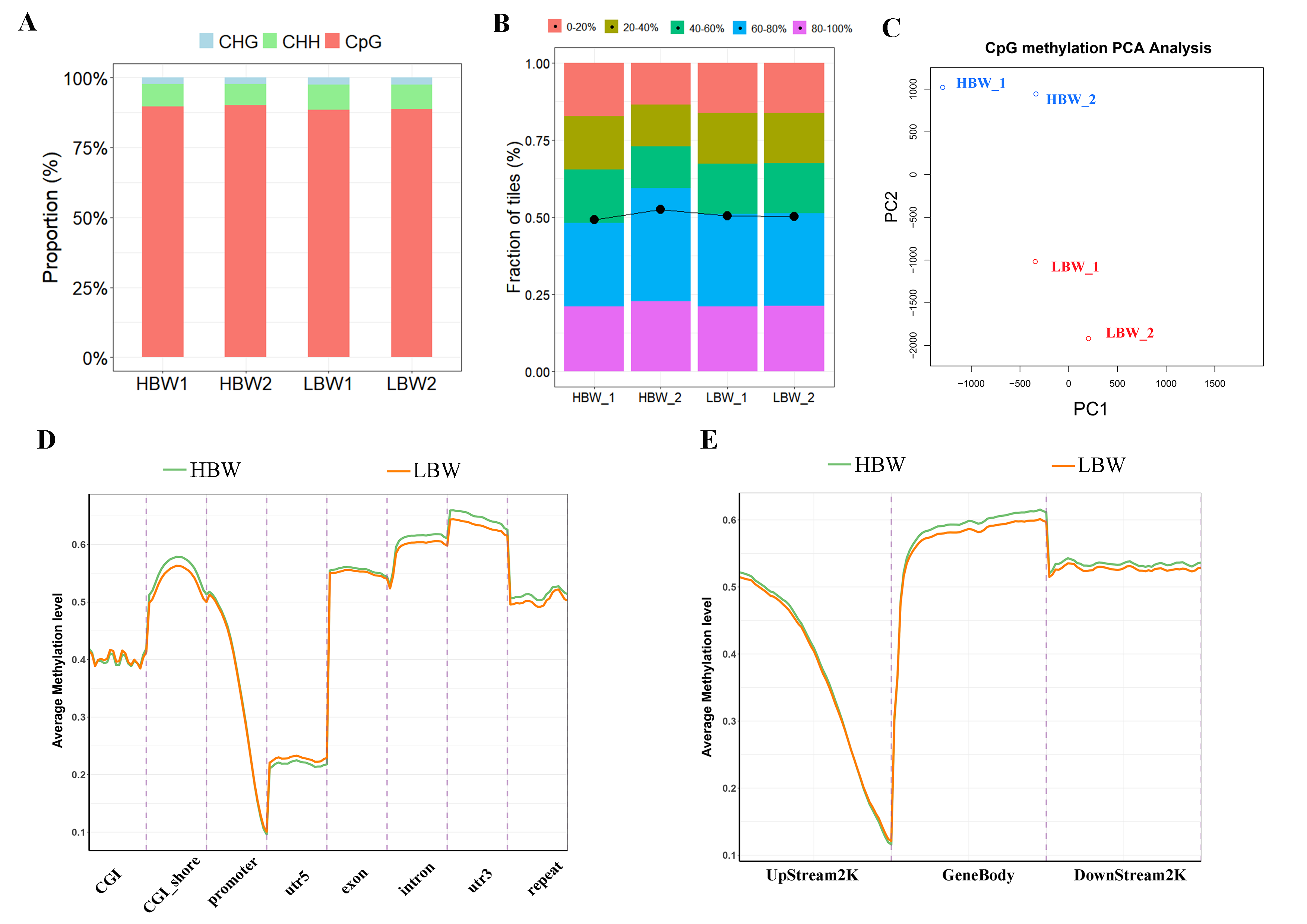

Supplement: Supplementary file 1 [file ijms-25-07702-s001.zip › Figure/Figure S3.tif]

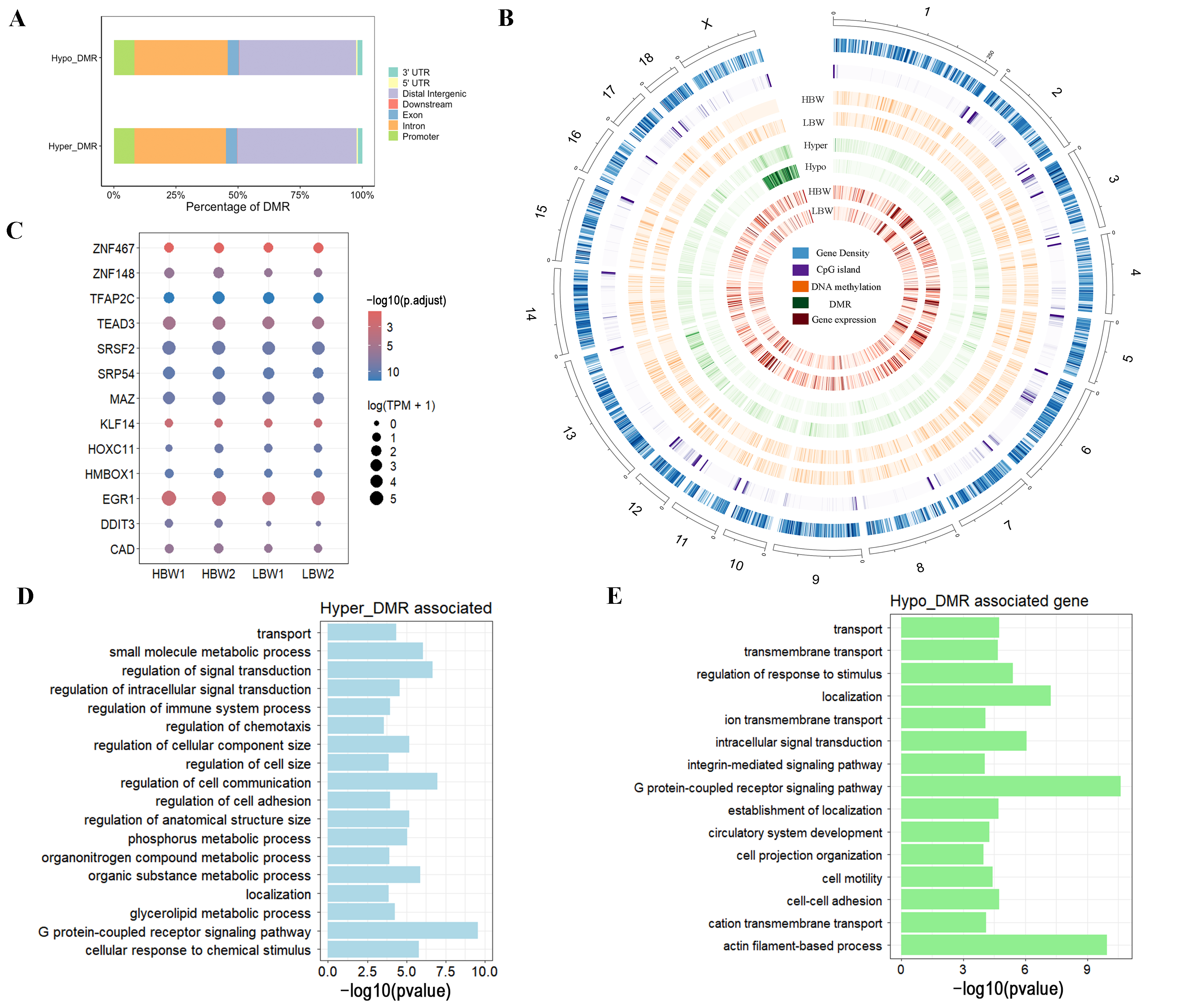

Supplement: Supplementary file 1 [file ijms-25-07702-s001.zip › Figure/Figure S4.tif]

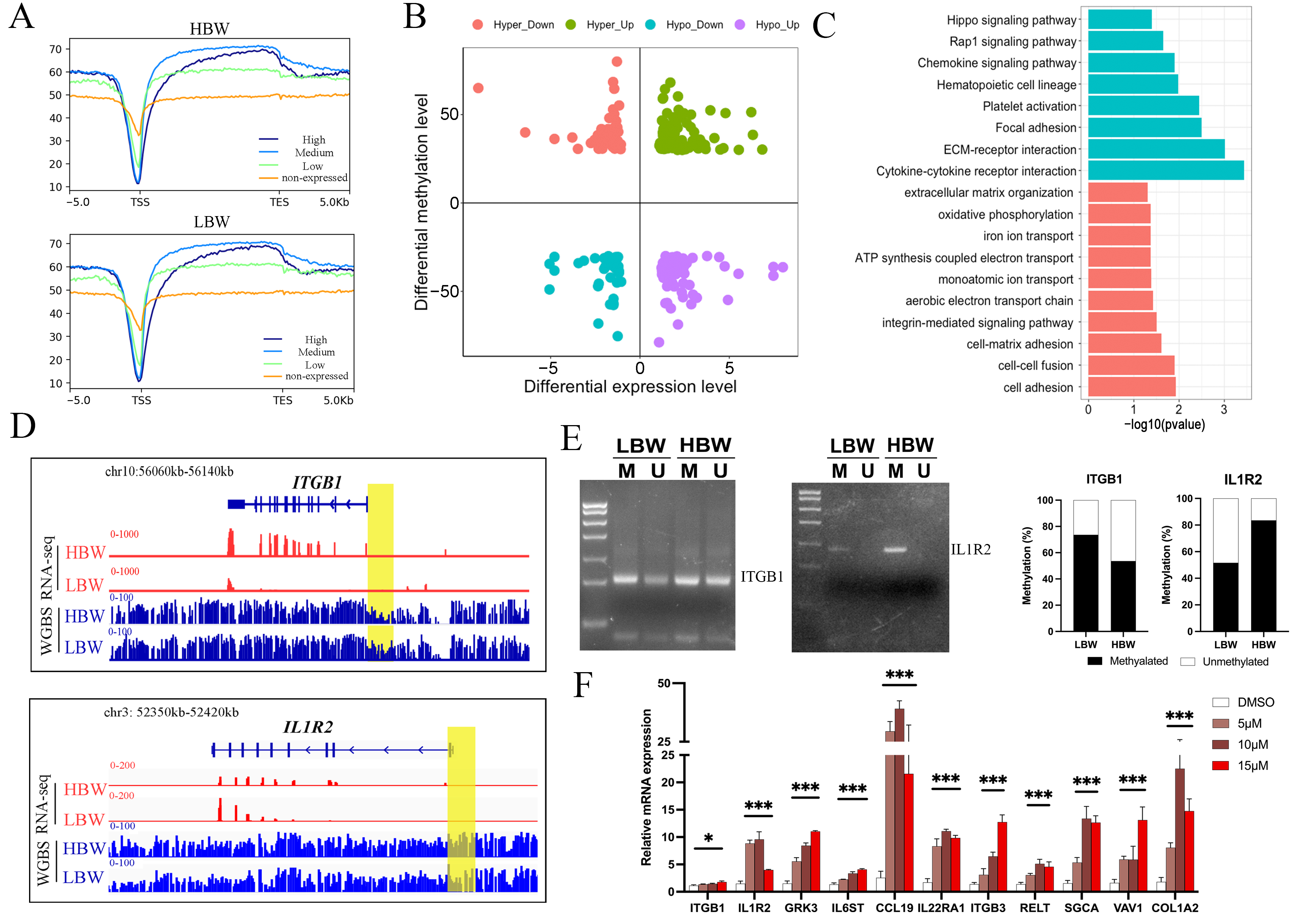

Supplement: Supplementary file 1 [file ijms-25-07702-s001.zip › Figure/Figure S5.tif]
